# Topical treatment of chemotherapy-induced peripheral neuropathy (CIPN) with high-concentration (179 mg) capsaicin patch in breast cancer patients – results of the QUCIP study

**DOI:** 10.3389/fonc.2024.1452099

**Published:** 2024-09-06

**Authors:** Michael Patrick Lux, Lilit Flöther, Catrin Frömter, Brigitte Rack, Kristina Veselinovic, Myriam Heine, Stefan Paepke, Petra Krabisch, Tamara Quandel, Rainer Sabatowski

**Affiliations:** ^1^ Department for Gynecology and Obstetrics, St. Louise Women’s Hospital, Paderborn, St. Josefs Hospital, Salzkotten, St. Vincenz Clinics Salzkotten & Paderborn, Paderborn, Germany; ^2^ Anesthesiology and Surgical Intensive Care, University Hospital Halle (Saale), Halle, Germany; ^3^ Clinic for Gynecology and Obstetrics, St. Georg Hospital Leipzig, Leipzig, Germany; ^4^ Gynecology and Obstetrics, University Hospital Ulm, Ulm, Germany; ^5^ Grünenthal GmbH, Medical Affairs D-A-CH, Aachen, Germany; ^6^ Gynecology Clinic, Hospital rechts der Isar, Technical University of Munich, Munich, Germany; ^7^ Gynecology and Obstetrics, Hospital Chemnitz, Chemnitz, Germany; ^8^ Pain Clinic, Department of Anesthesiology and Intensive Care, Medical Faculty “Carl Gustav Carus”, Technical University, Dresden, Germany

**Keywords:** high-concentration capsaicin, painful chemotherapy-induced peripheral neuropathy, CIPN, non-interventional study, topical therapy, peripheral neuropathic pain, quality of life, breast cancer

## Abstract

**Background:**

Chemotherapy-induced peripheral neuropathy (CIPN) following oral or intravenous chemotherapy often results in neuropathic pain, accompanied by symptoms such tingling, burning and hypersensitivity to stimuli, with a notable decline in quality of life (QoL). Effective therapies for CIPN are lacking, with a high demand for analgesics to address this issue. The QUCIP study aimed to assess the effectiveness of high concentration (179 mg) capsaicin patch (HCCP) in alleviating neuropathic pain and associated symptoms in breast cancer patients with confirmed CIPN.

**Methods:**

QUCIP is a prospective, multi-center observational study spanning 36 weeks with up to three HCCP treatments. Initial treatment (visit V0) was followed by two telephone contacts (T1, T2) and subsequent face-to-face visits every 12 weeks or upon retreatment (visits V1–V3). 73 female patients with painful CIPN post neoadjuvant/adjuvant breast cancer therapy were enrolled. Primary endpoint was the reduction of neuropathic pain symptom score (painDETECT^®^). Secondary endpoints included improvements in CIPN-specific QoL (QLQ-CIPN20), reductions in pain intensity (numeric pain rating scale, NPRS), and achievement of ≥ 30% and ≥ 50% pain reduction.

**Results:**

Median age was 61 years, with 52.0% of patients experiencing peripheral neuropathic pain for > 1 year (> 2 years: 34.2%). The painDETECT^®^ score significantly decreased from baseline (19.71 ± 4.69) to 15.80 ± 6.20 after initial treatment (p < 0.0001) and continued to decrease at follow-up visits. The NPRS indicated significant pain intensity reduction at each time point, particularly pronounced in patients receiving three HCCP treatments. Clinically significant pain relief of ≥ 30% increased from 25.0% at week 4 (T2) to 36.2%, 43.5%, and 40.0% at weeks 12 (V1), 24 (V2), and 36 (V3), respectively. The percentage of patients achieving pain relief of ≥ 50% increased from 14.7% at T2 to 15.5%, 21.7% and 32.5% at V1, V2 and V3, respectively. Patients further reported a significant improvement in their CIPN-related QoL throughout the study. Adverse drug reactions (ADRs) mainly included application site reactions.

**Conclusion:**

In this study, HCCP shows benefit in managing CIPN in real-world settings. The data demonstrate a sustained and progressive reduction in neuropathic pain and symptomatology, confirming the clinical benefit of repeated treatment observed in former clinical trials. HCCP treatment has also the potential to significantly improve the QoL associated with CIPN. The safety profile of HCCP was confirmed, supporting its use in clinical practice.

## 1 Introduction

Neuropathic pain, as defined by the International Association for the Study of Pain (IASP), results from a lesion or disease of the somatosensory nervous system, including both peripheral and central components. It is characterized by diminished sensory function or perception, together with increased sensitivity to pain or the occurrence of spontaneous pain (1, 2). Chemotherapy-induced peripheral neuropathy (CIPN) is one of the most common and distressing side effects of oral or intravenous chemotherapy. Patients undergoing chemotherapy may experience tingling, numbness, burning sensations, and sharp shooting pain in their hands and feet (3). As the treatment progresses, these symptoms can worsen, making everyday tasks a challenge. Balance issues, difficulties in walking, and fine motor skill impairment are also common, significantly impacting patients’ quality of life (QoL) (4, 5). Moreover, CIPN can lead to dose limitation or discontinuation of cancer therapy (6).

Agents that commonly cause CIPN include taxanes, platinum derivatives, vinca alkaloids, thalidomide, and proteasome inhibitors. CIPN can occur after the first dose of chemotherapy and manifests clinically as sensory, motor and/or autonomic deficits of varying severity. However, sensory symptoms often predominate, usually presenting as glove-like or stocking-like sensations in the hands and feet, and possibly in the face (7). The prevalence and severity of CIPN depends on several factors, including the chemotherapeutic drug (or combination of drugs) used, the cumulative dose, the duration of exposure as well as patient-specific risk factors (e.g. pre-existing neuropathy, age, and genetic factors) (8, 9). In general, the prevalence of CIPN decreases over time after cessation of treatment. Meta-analysis data showed an overall prevalence of CIPN across most chemotherapy and cancer types of 68% in the first month after the end of chemotherapy, 60% after three months and 30% after six or more months after the end of treatment, indicating persistence of CIPN in about 1/3 of patients (10). In the treatment of breast cancer, taxanes and platinum derivatives are frequently used (6). A systematic review found that despite appropriate therapy, 11% to over 80% of women with early-stage breast cancer experience persistent CIPN for one to three years after treatment initiation (11). In addition, 28% still experience moderate to severe CIPN symptoms on average 5.6 years after their last chemotherapy treatment (12).

Managing CIPN is still a significant challenge for healthcare professionals and the potential for work-related disability further contribute to a considerable economic strain on the healthcare system. There are currently no specific medical treatments to prevent or reduce the risk of developing CIPN. So far, only exercise or extremity cooling may be considered (9, 13–15). Existing treatments primarily focus on pain relief and symptom management, typically involving medications such as anticonvulsants, antidepressants and opioids (9, 16). However, these drugs are often associated with systemic adverse side effects such as dizziness and somnolence and may not provide adequate relief. Consequently, based on the available evidence, the 2020 American Society of Clinical Oncology (ASCO) guideline update does not recommend any specific agents for the treatment of CIPN, aside from considering the potential use of duloxetine (17, 18). The guideline also emphasized the necessity for additional research on the topical application of capsaicin (18). In addition to pain medication, physical therapy and functional exercises (balance, sensorimotor, and fine motor skill training) are used to address mobility and functional limitations (9).

The high-concentration (179 mg) capsaicin patch (HCCP) is recommended by current guidelines as a second-line treatment for neuropathic pain (16, 19–21) and as a first-line option for localized neuropathic pain (16). Unlike systemic medication, HCCP acts locally at the site of application. In the European Union, it has been authorized for addressing all conditions of peripheral neuropathic pain in adults, either alone or alongside other pain medication (22). The active component of HCCP, capsaicin, is a highly selective agonist for the transient receptor potential vanilloid subtype 1 (TRPV1), a non-selective cation channel found on nociceptive C- and Aδ-fibres. Prolonged activation of TRPV1-expressing nociceptors by HCCP application leads to long-term pain relief through defunctionalization of cutaneous nociceptors, rendering them less sensitive to various stimuli (23, 24). The induced changes are reversible, with restoration of normal function observed in healthy subjects within a few weeks (25). Treatment can be repeated every 90 days and, if necessary, with a minimal interval of 60 days (22). Several studies indicate that repeated treatments result in an increased response rate (26–30). Of particular note is the observation that in cases of inadequate pain relief after initial treatment, subsequent treatments can produce responses similar to those seen in initial responders, e.g. in terms of reduced pain intensity and improved sleep quality (31).

HCCP has been shown to reduce pain in several clinical and real-world studies in peripheral neuropathies of various etiologies (28–30, 32–37). The effectiveness has been demonstrated to be similar to orally administered centrally acting agents, while offering the advantage of minimal systemic side effects and no known drug-drug interactions (22, 35, 38). This reduces the risk of complications and is especially relevant for patients with polymedication and for multimorbid patients and/or patients undergoing cancer therapy. In a systematic review, Cabezón-Gutiérrez et al. conclude that HCCP can provide significant pain relief in patients with CIPN, but the authors criticize the small number of relevant studies (39).

In a subgroup of the non-interventional QUEPP study, promising results were observed regarding HCCP treatment in CIPN. Among 15 patients experiencing painful CIPN, 47% (n=7) reported ≥ 30% pain reduction, while 33% (n=5) experienced a pain reduction of ≥ 50% upon HCCP use (13). These findings suggest a similar effectiveness of HCCP in CIPN patients when compared to the overall study population, which consisted of 1,044 patients with various neuropathic pain conditions (32). In addition, a single-center study indicates that HCCP was an effective method for alleviating pain in 18 patients with CIPN caused by oxaliplatin (40). A more recently published monocentric study in 16 patients with CIPN provided further evidence of the significant pain reduction achieved with the use of HCCP. The study also suggested that this treatment approach may have disease-modifying effects by promoting sensory nerve fiber regeneration and phenotypic restoration. Within three months after HCCP treatment, the application led to a notable increase and normalization of marker proteins associated with intraepidermal and subepidermal nerve fibers, as determined by evaluation of skin biopsies (41). Capsaicin-mediated nerve regeneration has also recently been reported in the context of cold-induced neuropathic pain associated with non-freezing cold injury (NFCI) and diabetic peripheral neuropathy (42, 43). This regeneration of nerve fibers seemed to correlate with pain relief (41–43). Similarly, recent data indicate a correlation between significant pain reduction and functional regeneration of peptidergic nociceptors four weeks after HCCP treatment based on heat-induced neurogenic vasodilation supporting the disease-modifying effect of capsaicin (44). A retrospective chart review study consisting of 57 patients with CIPN revealed significant or complete pain relief upon 33.2% of HCCP applications corresponding to 43.9% of the patients. The trial identified several factors that influenced the effectiveness of the treatment. In particular, the type of underlying chemotherapy was relevant, with platinum-containing regimens showing less benefit than other types of chemotherapy. In addition, the line of analgesic treatment demonstrated an impact, with second-line use exhibiting significantly higher efficacy compared to third-line use. Finally, the effectiveness of HCCP treatment significantly improved with each subsequent treatment (26).

Currently, there is a lack of evidence-based effective therapies or preventive measures for the treatment of CIPN. The QUCIP study aimed to address this critical gap by providing new insights into the effectiveness of HCCP in reducing neuropathic pain and symptom severity. This open-label, non-interventional, observational study focused on breast cancer patients with clinically confirmed CIPN. Conducted under real-world clinical conditions, the study also assessed the impact of HCCP on patients’ QoL. Additionally, it evaluated the benefit of up to three HCCP treatments over a 9-month follow-up period, providing valuable data on the potential cumulative effectiveness of multiple treatments. The results of this study will be valuable in advancing therapeutic strategies and optimizing clinical management protocols for CIPN.

## 2 Materials and methods

### 2.1 Study design and participants

QUCIP is a prospective, non-interventional, multi-center, observational study conducted at 7 sites in Germany with a follow-up period of 36 weeks (9 months) and up to 3 treatments with HCCP. The study was conducted in compliance with the German Drug Law (§ 67.6 AMG). QUCIP was approved by the Ethics Committees of the Technical University of Munich (approval number: 610/19 S-SR), the University Hospital of Ulm (approval number: 440/20 – CL/TR), and the Ethics Commission of the State Chamber of Medicine in Sachsen for the Faculty of Medicine at the Technical University of Dresden and the St. Georg Hospital Leipzig (approval number: EK-BR-64/21/1). Detailed information about the study was given to all participants and written informed consent was obtained from all subjects before the study started. All aspects of the study were performed in accordance with the Declaration of Helsinki.

Adult patients (between 18 and 90 years of age) with painful CIPN after neoadjuvant/adjuvant breast cancer therapy with taxane- and/or platinum-based chemotherapeutic drugs who were naïve for HCCP and had a painDETECT^®^ score ≥ 13 were eligible for inclusion. Patients included in the study should have intact skin in the area to be treated. Patients still during chemotherapy or who were undergoing palliative chemotherapy, were pregnant, or had contraindications according to the HCCP Summary of Product Characteristics (SmPC) (22) or insufficient knowledge of German were excluded from participation.

**Figure 1:**
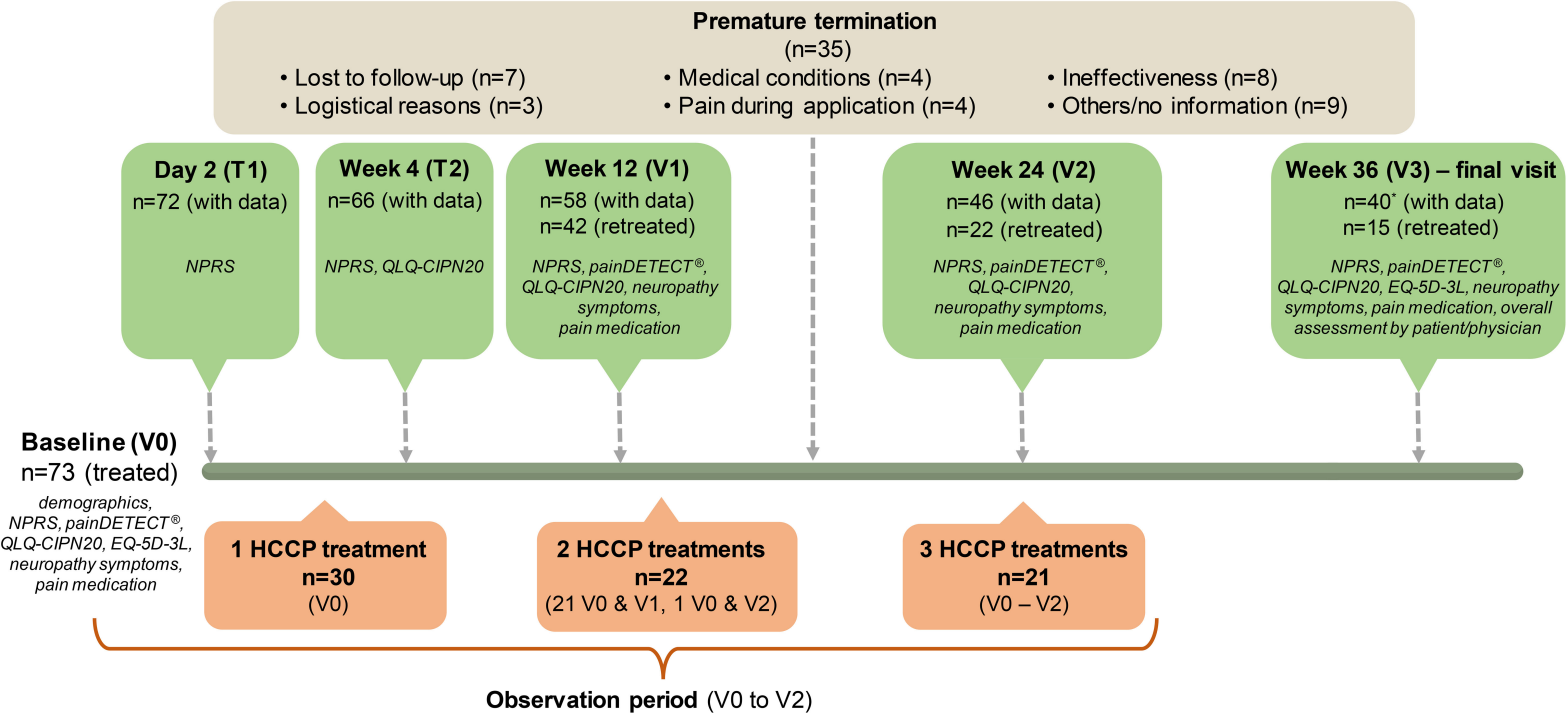
Flowchart of study participant disposition and data collection. Visit 3 was the end of the study. Therefore, only results from treatments between V0 and V2 were analysed. During this period, 30 patients received one treatment, 22 received two treatments and 21 patients underwent all three treatments. Frequency, seriousness, and severity of ADRs were recorded throughout the observational period.

Following the initial application of HCCP (visit 0, V0), two telephone contacts (T1, T2) were conducted after 2 days and 4 weeks. Subsequent face-to-face visits took place every 12 weeks and/or at the time of a possible retreatment (visits V1–V3) (**Figure 1**).

### 2.2 Study endpoints and data collection

The primary objective was to evaluate the change in neuropathic pain symptomatology after a single treatment with HCCP as compared to baseline by means of the painDETECT^®^ questionnaire. The questionnaire’s final score was used to assess neuropathic pain and symptoms as the primary endpoint at visit V1 and secondary endpoints at later time points (V2 and V3).

Additional secondary endpoints comprised reduction of pain intensity using an 11-point numeric pain rating scale (NPRS), pain reduction of ≥ 30% and ≥ 50%, CIPN-specific QoL (Quality of Life Questionnaire-CIPN twenty-item; QLQ-CIPN20), health-related QoL (European Quality of Life 5 Dimensions 3 Level; EQ-5D-3L), satisfaction with therapy by patients and physicians, and reduction in concomitant pain medication. Secondary endpoints of the study also included the tolerability, i.e. assessment of frequency, seriousness, and severity of adverse drug reactions (ADRs).

Baseline data included demographics, length of chemotherapy, time since start of chemotherapy, and type of medication used for chemotherapy (i.e. taxanes and/or platinum derivates) as well as the duration of neuropathic pain.

Specifically, clinical data documented at baseline and follow-up visits (as far as collected within the scope of the physician’s practice) included (**Figure 1**):

- PainDETECT^®^ questionnaire at baseline and week 12, 24 and 36
- NPRS within last 24 hours and last 4 weeks at baseline and week 4, 12, 24 and 36
- QLQ-CIPN20 questionnaire at baseline and week 4, 12, 24 and 36
- Regular concomitant medication taken for neuropathic pain (substance names, daily dose) and regular non-pharmacological pain therapy at baseline and week 12, 24 and 36
- Questions regarding the presence of pre-specified neuropathic pain symptoms (i.e. allodynia, hyperalgesia, paresthesia, hypoalgesia, numbness, thermal hypoesthesia) asked at baseline and changes from baseline reassessed at week 12, 24 and 36 (% of patients with vanished, improved, unchanged or worsened symptoms)
- Tolerability, i.e. ADRs recorded at all visits and throughout the observation period
- Time to retreatments/intervals between treatments at week 12, 24 and 36
- EQ-5D-3L questionnaire at baseline (V0) and at the final visit (V3)
- Self-assessment of treatment by patient and physician at V3 (% patients or physicians assessing treatment as good, moderately good, sufficient, or bad)

### 2.3 Questionnaires used in the study

#### 2.3.1 PainDETECT^®^

The painDETECT^®^ questionnaire, which has been validated for the detection of neuropathic pain, provides a *final score* of 0–38 from nine questions covering the frequency and quality of seven neuropathic symptoms, pain pattern over time, and radiating pain (45). Individual scoring of the seven neuropathic symptoms on a scale of 0–5 (never to very severe) results in a *total score* of 0–35 and allows changes in the intensity of sensory symptoms to be tracked over the course of therapy (46, 47). A painDETECT^®^ final score of ≥ 19 implies that a neuropathic component is likely (> 90%), a score of 13-18 suggests possible neuropathic pain, and a score of ≤ 12 considers that a neuropathic component is unlikely (< 15%).

#### 2.3.2 QLQ-CIPN20

This patient self-report questionnaire supplements the core QoL questionnaire of the European Organization for Research and Treatment of Cancer (EORTC), which has been validated for use in German cancer population (48, 49). The QLQ-CIPN20 is a 20-item questionnaire evaluating sensory, motor, and autonomic symptoms associated with CIPN and their impact on function with each item measured on an ordinal 1-4 scale (1, not at all; 4, very much). Accordingly, the evaluation of the QLQ-CIPN20 questionnaire yields 3 different subscales for sensory, motor, and autonomic impairment in QoL. All scores are converted linearly to a scale of 0–100, with higher scores indicating greater symptom burden. The data are related to the experiences of the previous week. As the item “Did you have difficulty getting or maintaining an erection” (Item 20) did not apply to the female patients in this study, only Items 1-19 were utilized.

#### 2.3.3 EQ-5D-3L

The EQ-5D-3L is a general tool for measuring QoL independent of disease and includes the EQ-5D descriptive system as well as the EQ visual analogue scale (EQ-VAS, 0-100) (50, 51). A validated German version was used (52). The descriptive system comprises five dimensions: mobility, self-care, usual activities, pain/discomfort, and anxiety/depression, each with three levels. Health-related QoL was quantified in relation to population-standardized reference values (EQ-5-Health Utility Index) ranging from -0.594 (indicating the worst possible health status) to 1 (reflecting full health status) (53).

### 2.4 Statistical methods and data analysis

The analysis of the collected data was performed descriptively. Continuous data were described using mean, median, standard deviation (SD), quartiles, minimum, and maximum. Changes between baseline (V0) and the follow-up visits (V1–V3) were tested using the two-sided one-sample t-test. The primary endpoint was additionally analyzed using the variance analysis with repeated measurements. Categorical parameters were presented as absolute and relative frequencies. The sample size was determined in the statistical analysis plan (SAP) based on the primary endpoint and the requirements for a two-sided t-test for equality of means in matched pairs. For the calculation, we assumed a 2-point reduction in the painDETECT^®^ final score, a standard deviation of 5 points, a significance level (α) of 0.05 (2-sided), and a power of 90% (54).

All patients were treated in at least one of their neuropathic pain areas and finally evaluated (intention-to-treat set) as far as documented. The full analysis set comprised 73 patients. Analyses were performed for the actual number of evaluated patients for whom the outcome was obtained for the respective visits, i.e. observed case (OC) analysis and/or for patients for whom values were available for all visits (V1–V3, completer analysis) as indicated in the manuscript. The SAS^®^ 9.4 (TS1M6) statistical software was used to generate all tables, figures, listings, and statistical analyses.

## 3 Results

### 3.1 Baseline characteristics and HCCP treatments

Data analysis includes 73 patients treated at least once with HCCP (**Table 1**). Median age in the study cohort at baseline was 61 years (36–80 years). Patients had received various taxane- and/or platinum-based chemotherapeutic agents; however, the majority was treated among others with paclitaxel. The duration of chemotherapy was 172.1 ± 173.9 (mean ± SD) days (median: 143 days) and the mean duration between end of chemotherapy and enrolment into the study was 2.4 ± 2.3 (mean ± SD) years (median: 1.6 years) prior to the individual observation start. In 52.0% of the patients, the peripheral neuropathic pain had lasted for more than one year (> 2 years: 34.2%).

**Table 1:** Baseline characteristics of CIPN patients included in the QUCIP study.

Characteristic	Patients with CIPN (n=73)
Age (mean ± SD, median, [range])	61.5 ± 10.2 years, 61 years [36–80 years]
End of chemotherapy (prior to study start, mean ± SD, median, [range])	2.4 ± 2.3; 1.58 years [0.04-9.64 years]
Chemotherapeutic agents*	Number of patients, n
*Paclitaxel*	*60*
*Nab^1^-Paclitaxel*	*2*
*Docetaxel*	*7*
*Other taxane derivative*	*5*
*Carboplatin*	*14*
Duration of chemotherapy (mean ± SD, median)	172.1 ± 173.9; 143 days
Duration of peripheral neuropathic pain	Number of patients, n (%)
*3 – 12 months*	*35 (48%)*
*> 1 – 2 years*	*13 (18%)*
*> 2 years*	*25 (34%)*
Neuropathic symptoms (asked about apart form painDETECT^®^ at baseline)*	Number of patients, n (%)
*Allodynia*	*17 (23%)*
*Hyperalgesia*	*30 (41%)*
*Hypoalgesia*	*23 (32%)*
*Paresthesia*	*52 (71%)*
*Numbness*	*63 (86%)*
*Thermhypoesthesia*	*27 (37%)*
*Thermhyperesthesia*	*32 (44%)*
*Other*	*15 (21%)*
Patients with concomitant medications for neuropathic pain*, n (%)	26 (36%)
*1 concomitant medication*	*20 (27%)*
*2 concomitant medications*	*5 (7%)*
*3 concomitant medications*	*1 (1%)*
*at least 1 antidepressant^2^*	*1 (1%)*
*at least 1 anticonvulsant^3^*	*11 (15%)*
*at least 1 opioid^4^*	*1 (1%)*
*Others^5^*	*14 (3%)*
Area treated (baseline)	Number of patients, n (%)
*Hand/hands*	*5 (7%)*
*Foot/feet*	*33 (45%)*
*Hand/hands & foot/feet*	*35 (48%)*
Number of patients discontinuing before end of study (9 months), n (%)	35 (48%)

*Multiple answers were possible; ^1^Nab: nanoparticle albumin bound; ^2^amitriptyline, duloxetine. ^3^gabapentin, pregabalin; ^4^hydromorphone; ^5^non-steroidal anti-inflammatory drugs (ibuprofen, diclofenac), metamizole/dipyrone, and nutritional supplements.

Most patients were treated for their peripheral neuropathic pain at the foot or feet (n=33) and/or at both, hand/hands and foot/feet (n=35). Pain treated solely in the hand/hands was less frequently reported (n=5).

At baseline, a significant number of patients reported numbness (n=63, 86%) and paraesthesia (n=52, 71%) as their primary symptoms, followed by thermal hyperesthesia, hyperalgesia, thermal hypoesthesia, hypoalgesia, and allodynia (**Table 1**). At least one stable concomitant medication for neuropathic pain was reported in 35.6% of patients (n=26).

**Figure 1** illustrates the patient flow, including the number of treatments during the follow-up period and the assessments carried out at baseline, via telephone contact and during visits. According to the protocol, up to three HCCP follow-up treatments per patient per treatment area were possible at the scheduled visits over the study duration. 30 patients were treated once, 22 patients twice and 21 patients three times during the observation period (V0 to V2). 15 patients were retreated at V3, results of this treatment were not part of the study evaluation. The median time from the initial to the second treatment was 91 days (range: 70 to 149; n=21), with a corresponding median duration of 91 days between the second and third treatment (range: 62 to 168; n=12). The entire study period (9 months) was completed by 38 patients. For two dropouts (after V0 and V1, respectively), overall assessment of the treatment was documented at V3.

A total of 35 patients discontinued the study prematurely (15 patients after V0, 12 patients after V1 and 8 patients after V2). Discontinuation was initiated by the physician in three cases and by patients in 25 cases. Seven participants were “lost-to-follow-up” (e.g. could not be contacted by phone and did not show up at visits). Reasons for discontinuation included logistical issues (n=3; e.g., desire for treatment closer to home), medical conditions (n=4; metastases, palliative chemotherapy, or other health problems), ineffectiveness (n=8, with 2 including logistical reasons and 1 including pain at the application site), and pain during application (n=4). Other reasons (n=9) included changing physicians, transitioning to non-pharmacological treatment, and lack of information.

### 3.2 Neuropathic pain and sensory symptoms

**Figure 2:**
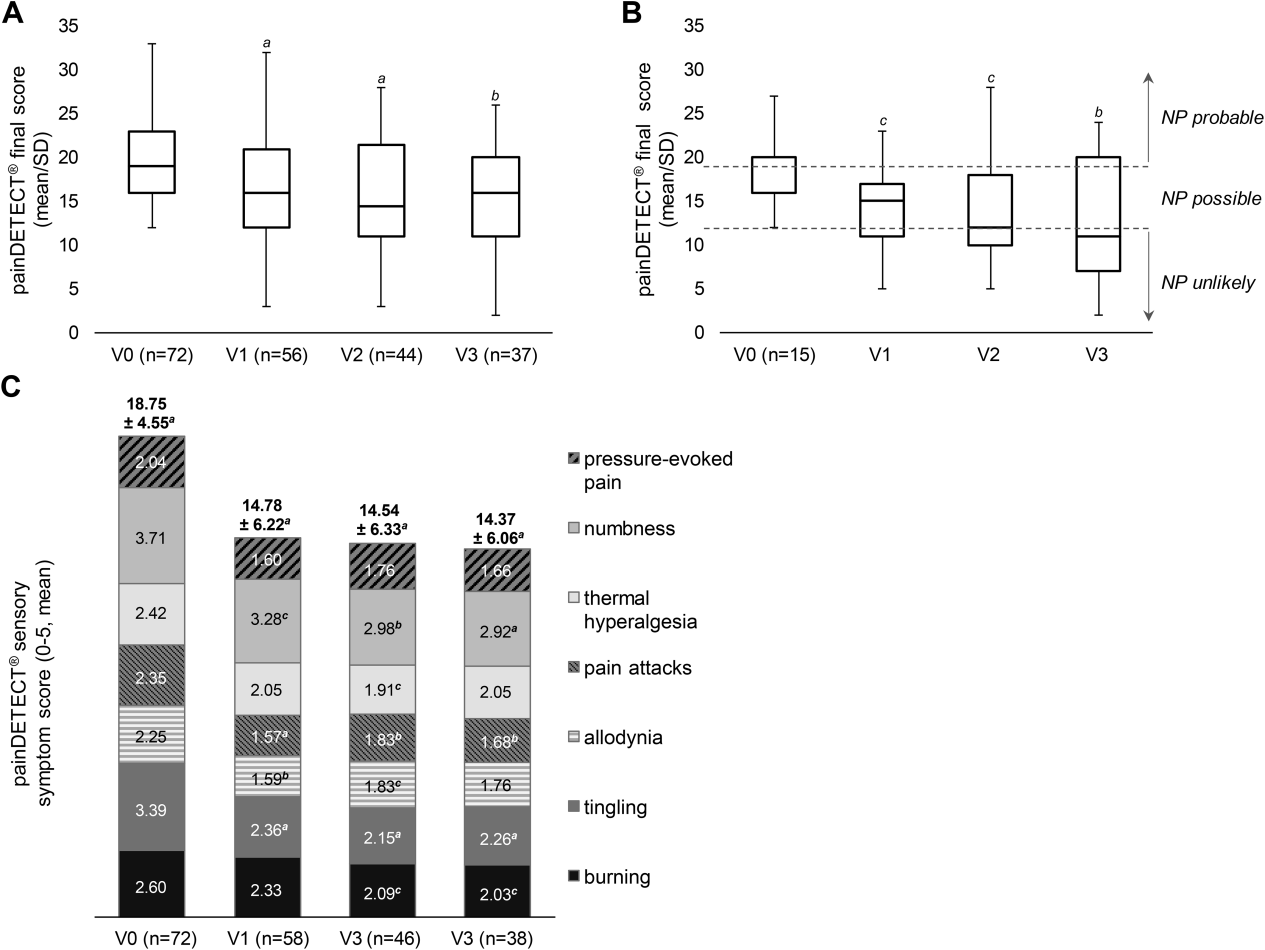
Reduction in painDETECT^®^ scores. **(A, B)** Mean change in painDETECT**^®^** final score (scale 0–38) based on OC data **(A)** and on data from patients who received 3 consecutive HCCP treatments at V0, V1, and V2 **(B)**. After three treatments patients achieved average final painDETECT^®^ scores below 12, indicating a reduced likelihood of neuropathic pain. **(C)** Mean change in painDETECT**^®^** total score based on the assessment of seven sensory symptoms (score 0–35, above pillars) as well as of individual symptoms (scale from 0-5). NP: neuropathic pain. SD: standard deviation. V: visit. *^a^* p ≤ 0.001, *^b^* p ≤ 0.01, *^c^* p ≤ 0.05 vs baseline (two-sided one-sample t-test). No separate median line is shown for V0 in **(B)** because the median coincides with the first quartile.

All patients had a diagnosis of CIPN from the treating physician. Primary endpoint was the painDETECT^®^ final score at V1 compared to V0 at baseline. The presence of probable neuropathic pain is defined by a final score of ≥ 19, which was observed at V0 as a median by 72 patients (**Table 2**). Based on observed data, the average painDETECT^®^ final score was 19.71 ± 4.69 at baseline and significantly decreased to 15.80 ± 6.20 at V1. The difference of -4.0 points was statistically highly significant (p < 0.0001), suggesting a potentially clinically relevant improvement. A statistically significant decrease of the painDETECT^®^ final score from baseline was also observed at all follow-up visits (V2 and V3) (**Figure 2A**).

**Table 2:** Neuropathic pain and impact on QoL according to painDETECT^®^, NPRS and QLQ-CIPN20.

Measure	V0	V1	Change	p value*
PainDETECT^®^ final score, mean (SD)	19.71 (4.69) (n=72)	15.80 (6.20) (n=56)	-4.00 (6.05)	< 0.0001
PainDETECT^®^ total score, mean (SD)	18.75 (4.55) (n=73)	14.78 (6.22) (n=58)	-3.89 (6.00)	< 0.0001
Average pain intensity (NPRS, last 24 h), mean (SD)	6.23 (1.89) (n=71)	5.01 (2.19) (n=58)	-1.21 (2.08)	< 0.0001
QLQ-CIPN20 total score, mean (SD)	45.01 (18.43) (n=73)	37.07 (18.42) (n=57)	-7.63 (13.08)	< 0.0001
Sensory subscale, mean (SD)	49.33 (19.64)	41.07 (19.51)	-7.60 (14.94)	0.0003
Motor subscale, mean (SD)	45.50 (24.71)	36.68 (22.93)	-8.69 (18.38)	0.0007
Autonomic subscale, mean (SD)	23.84 (26.95)	20.47 (24.20)	-4.09 (19.74)	0.1231

SD, standard deviation; NPRS, numerical pain rating scale.

Change from visit 0 (V0) to visit 1 (V1). Analysis of variance with repeated measures. *two-sided one-sample t-test.

A total of 21 patients completed the study as planned in the observation plan, i.e. per protocol, and received 3 consecutive treatments with HCCP at visits 0, 1, and 2. Of these, 15 patients have complete follow-up documentation for the painDETECT^®^ final score. Assessment of this patient cohort revealed a consistent and significant decrease in the painDETECT^®^ final score from baseline across all follow-up visits, indicating steady improvement with each successive treatment. At V3 the mean score reached a value below 12 (i.e. neuropathic pain considered unlikely) (**Figure 2B**).

The presence and grading of the seven neuropathic symptoms (i.e. burning sensation, tingling sensation, allodynia, pain attacks, thermal hyperalgesia, numbness, pressure-evoked pain) were significantly reduced according to the painDETECT® total score, which improved from 18.75 ± 4.55 at V0 to 14.78 ± 6.22 at V1 (p < 0.0001) (**Table 2**). The score also remained significantly reduced compared to baseline at V2 and V3 (p < 0.001) (**Figure 2C**). The most prominent symptoms according to the painDETECT^®^ questionnaire at baseline were tingling and numbness, followed by burning sensation, pain attacks, and thermal hyperalgesia. Allodynia and pressure-evoked pain were less pronounced. Significant improvements were observed at V1 for tingling, allodynia, pain attacks, and numbness. At visit V2 and/or V3, significant improvement was noted for all neuropathic symptoms except for pressure-evoked pain (**Figure 2C**).

Changes in neuropathic symptoms reported by the patients at baseline in addition to the painDETECT^®^ questionnaire, including allodynia, hyperalgesia, paresthesia, hypoalgesia, numbness, thermal hypoesthesia, and thermal hyperesthesia (**Table 1**), were reassessed at V1 to V3. The results demonstrated a progressive improvement in specific symptoms over the course of the study. At V3, neuropathic symptoms had either completely disappeared or showed improvement in approximately 1/3 of the patients with available information (n=40). In particular, thermal hypoesthesia, thermal hyperesthesia, numbness, hypoalgesia, and allodynia showed the highest reductions at V3, with each symptom improving in 30.0% of patients, followed by hyperalgesia and paresthesia, which each improved in 25.0% of patients.

**Figure 3:**
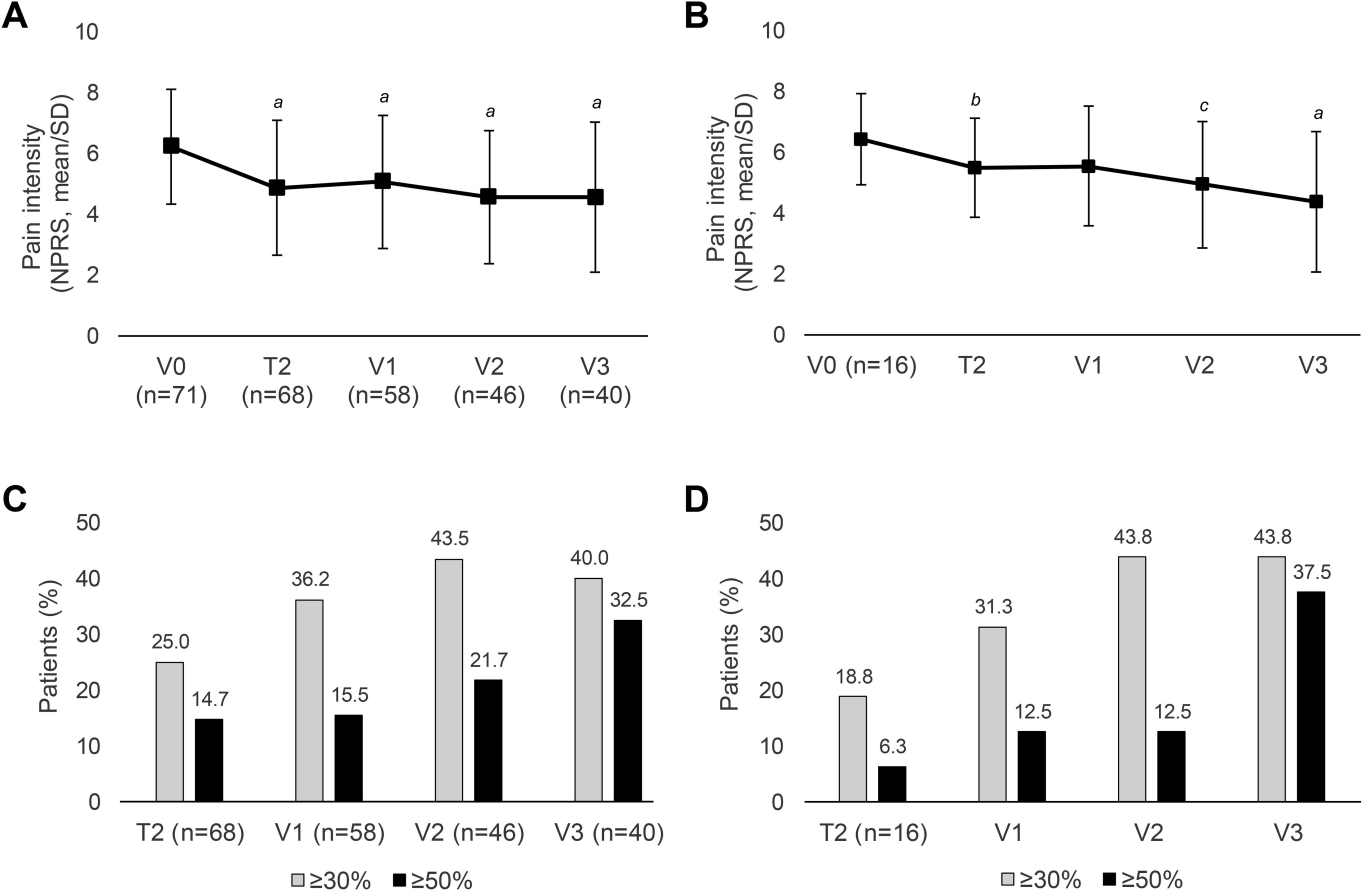
Course of peripheral neuropathic pain intensity and response rates. **(A, B)** Average daily pain intensity recorded by NPRS in the total cohort (OC) **(A)** and in patients who received three consecutive HCCP treatments at V0 to V2 **(B)**. **(C, D)** Percentage of patients with pain reduction of ≥ 30% or 50% in the total cohort (OC) **(C)** and in patients who received three consecutive HCCP treatments at V0 to V2 **(D)**. SD: standard deviation. T, telephone contact; V, visit. *^a^* p ≤ 0.001, *^b^* p ≤ 0.01, *^c^* p ≤ 0.05 vs baseline (two-sided one-sample t-test).

The average daily pain intensity recorded by NPRS significantly declined at V1 compared to V0 (**Table 2**) as well as at all follow-up visits (**Figure 3A**). Similarly, pain intensity over the last 4 weeks also improved significantly from V0 onwards at all visits compared to baseline (V1: -0.88 ± 2.29, n=57, p=0.0060; V2: -1.64 ± 2.28, n=45, p < 0.0001; V3: -1.18 ± 2.67, n=39, p=0.0090). Clinically meaningful pain reductions were explored using thresholds of ≥ 30% and ≥ 50% (32, 55). A response rate of ≥ 30% is considered a realistic goal in the treatment of neuropathic pain (16). The percentage of patients who experienced a reduction of at least 30% or 50% in their average daily pain compared to baseline showed a consistent increase throughout the study period. Three months after the second treatment (V2), there was a notable rise in the proportion of patients achieving a clinically meaningful pain reduction of ≥ 30%. Following the third treatment (V3), the proportion of patients classified as ≥ 50% responders particularly increased (**Figure 3C**).

Of the 21 patients who received three consecutive HCCP treatments (at V0-V2) every three months as outlined in the study protocol, 16 had complete follow-up pain intensity records (NPRS) for the last 24 hours. These patients experienced a sustained and progressively increasing reduction in mean daily pain intensity, which was statistically significant at every visit except of V1 (**Figure 3B**) and contributed to a steady increase in the proportion of patients experiencing clinically relevant pain reductions of ≥ 30% and ≥ 50% (**Figure 3D**).

The analysis of mean reduction in daily NPRS scores among patients with varying pain durations (≤ 6 months, 6 months to 2 years, and > 2 years) demonstrated a significant decrease in reported pain intensity at all visits compared to baseline (V0) within each category. The differences between V3 and V0 were -1.65 ± 1.77 (p=0.0002) for ≤ 6 months, -1.48 ± 2.89 (p=0.0171) for 6 months to 2 years, and -1.30 ± 1.84 (p=0.0026) for > 2 years.

### 3.3 Quality of life

**Figure 4:**
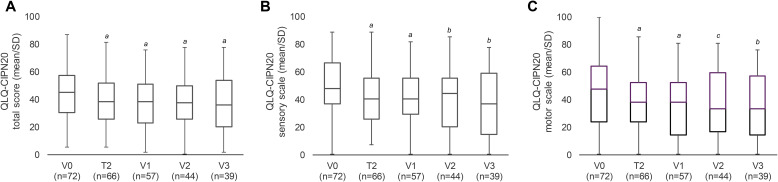
Reduction of CIPN-specific symptoms and improvement in QoL according to the QLQ-CIPN20 questionnaire. Mean change in QLQ-CIPN20 total score **(A)** and its sensory **(B)** and motor **(C)** subscales. Lower score corresponds to an improvement in QoL and a decrease in symptoms. Autonomous symptoms (not shown) remained mainly unchanged. SD, standard deviation; T, telephone contact; V, visit. *^a^* p ≤ 0.001, *^b^* p ≤ 0.01, *^c^* p ≤ 0.05 vs baseline (two-sided one-sample t-test).

In general, the reduction in pain intensity was accompanied by an improvement in the patients’ QoL throughout the study as assessed by QLQ-CIPN20. Autonomic symptoms were less pronounced at baseline (subscale: 23.84 ± 26.95) compared to sensory (subscale: 49.33 ± 19.64) and motor (subscale: 45.50 ± 24.71) symptoms. There was a significant improvement of the QLQ-CIPN20 total score from baseline at all visits (**Table 2**; **Figure 4A**). The sensory subscale of the QLQ-CIPN20 declined significantly from V0 to V1 by 7.60 points (p=0.0003) and the motor subscale by 8.69 points (p=0.0007) (**Table 2**; **Figures 4B, C**). These subscales were also significantly improved compared to baseline at all time points (**Figures 4B, C**). The autonomic scale remained largely unchanged over the course of the study with significant improvement only at T2 (17.93 ± 23.44, p=0.0129) and V2 (19.70 ± 24.71, p=0.0245).

The EQ-5D-3L questionnaire was completed at visits V0 and V3. An evident and consistent positive trend was observed in the dimensions of self-care, functionality, mobility, discomfort, and anxiety in the 40 patients who attended V3 (data not shown). Responses to the EQ-5D-3L descriptive profile were transformed into a single index score (utility index), demonstrating a significant enhancement at V3 in comparison to baseline for patients with available data at V0 and/or V3 (**Table 3**). Similar results were obtained for the subgroup of patients with data available at both time points (V0: 0.53 ± 0.31, V3: 0.69 ± 0.28, p=0.0095, n=38). The parameter “assessment of current health” (EQ-VAS, scale 0–100) also showed significant improvement at V3 (**Table 3**).

**Table 3:** Health-related quality of life (EQ-5D-3L)-weighted health status index (EQ-5-Health-Utility-Index) and assessment of current health (EQ-VAS).

Measure	V0 (n=73)	V3 (n=40)	Change	p value*
EQ-5-Health Utility Index, mean (SD)	0.56 ± 0.3	0.69 ± 0.27	0.15 ± 0.35	0.0107
EQ-VAS, mean (SD)	59.13 ± 18.12	65.64 ± 16.94	10.92 ± 16.31	0.0002

Change from visit 0 (V0) to visit 3 (V3). *two-sided one-sample t-test.

At the initial visit (V0), 35.6% of participants (26/73) used additional medication for peripheral neuropathic pain. This slightly increased to 37.9% (22/58) at the first follow-up (V1). By V2, a discernible decline was observed, with only 26.1% (12/46) of participants requiring concomitant analgesics. This pattern persisted at V3, where the proportion was 30.0% (12/40). Of the 26 patients using analgesics at V0, 10 were still using them at V3, six had stopped, and in 10 cases, no information was available. Two patients who were not taking any medication at V0 reported taking concomitant pain medication at V3.

### 3.4 Treatment evaluation by patients and physicians

At the end of observation (V3), the overall treatment assessment of the 40 patients remaining in the study was positive. 60.0% of patients (n = 24) rated their overall treatment with HCCP as “very good” or “good”, 22.5% (n=9) rated it as “moderate”, 7.5% (n=3) as “sufficient”, and 10.0% (n=4) as “poor”. Regarding pain outcomes, 50.0% (n=20) reported an “improved” or “much improved” condition, 42.5% (n=17) reported no change, and 7.5% (n=3) noted a “worsened” condition.

For 39 of the 40 patients, the physician’s assessment was available. From the physicians’ perspective, the treatment effect was rated as “very good” or “good” for 42.5% of their patients (n=17), “moderate” for 27.5% (n=11), “sufficient” for 15% (n=6), and “bad” for 12.5% (n=5).

Physicians assessed the tolerability of HCCP as “very good” or “good” for 70.0% of their patients (n=21) and 85% of the remaining 40 patients reported not experiencing side effects from the treatment.

In addition, both the integration into the clinical routine and the cost/benefit ratio were rated positively by most physicians. These parameters were rated as “very good” or “good” by 72.5% (n=29) and 55.0% of physicians (n=22), respectively.

### 3.5 Safety and tolerability

For 22 of the 73 patients at least one ADR was reported. A total of 34 ADRs were documented for these 22 patients (**Table 4**). The most frequently reported ADRs were application site pain (n=18, 24.7%), burning sensation (n=6, 8.2%), and application site erythema (n=3, 4.1%).

**Table 4:** Absolute and relative frequencies of ADRs according to System Organ Classes (MedDRA^®^ Version 26).

System Organ Class	Absolute frequency (n)	Relative frequency (%)*
Gastrointestinal disorders	2	
*Anorectal discomfort*	*1*	*1.37*
*Diarrhoea*	*1*	*1.37*
General disorders and administration site conditions	24	32.88
*Application site discomfort*	*1*	*1.37*
*Application site erythema*	*3*	*4.11*
*Application site pain*	*18*	*24.66*
*Application site paraesthesia*	*1*	*1.37*
*Drug ineffective*	*1*	*1.37*
Injury, poisoning and procedural complications	1	1.37
*Burns first degree*	*1*	*1.37*
Nervous system disorders	6	8.22
*Burning sensation*	*6*	*8.22*
Skin and subcutaneous tissue disorders	1	1.37
*Pruritus*	*1*	*1.37*
Total count of ADRs	34	
Total patients	22	

ADRs, adverse drug reactions; *Calculation based on overall number of patients (n=73).

Of the 34 ADRs, 2 ADRs (5.88%) were classified as serious and 32 ADRs (94.12%) as not serious. The two serious ADRs were reported as “application site erythema” and “application site pain” and both occurred in 1 patient. In terms of intensity, 6 of the 34 ADRs (17.65%) were classified as mild, 12 (35.29%) as moderate and 14 (41.18%) as severe. For 2 ADRs (5.88%), no information on intensity was provided.

## 4 Discussion

This non-interventional observational study aimed to monitor the effectiveness of HCCP in breast cancer patients with CIPN in the daily clinical practice over a 9-month study period with 2 telephone contacts (T1, T2) and 3 on-site visits (V1–V3) following the first treatment (at V0). The results of the effectiveness and tolerability assessments from the QUCIP study support HCCP as a valuable addition to the treatment options for patients with CIPN in routine clinical practice.

Although the painDETECT^®^ questionnaire is not validated for the longitudinal assessment of neuropathic pain, it provides valuable information on the evolution of neuropathic symptomatology over the course of treatment (46, 47). In this study, the final score of the painDETECT^®^ questionnaire demonstrated a significant reduction in neuropathic pain symptoms compared to baseline, including associated pain radiation and pain pattern at V1 (primary endpoint, **Table 1**) as well as at all follow-up visits (**Figure 1A**). Patients who received all 3 consecutive HCCP treatments within the observation period (i.e. at V0, V1, and V2) achieved a continuous reduction in the painDETECT^®^ final score. By V3, the score dropped below the value of 12 (neuropathic pain unlikely) indicating that HCCP treatment effectively reduces the clinical presentation of neuropathic pain (**Figure 1B**).

At baseline (V0), tingling and numbness were the predominant painDETECT^®^ sensory symptoms, followed by burning sensation, pain attacks, and thermal hyperalgesia, with less prominence for allodynia and pressure-evoked pain. V1 revealed significant improvements in tingling, allodynia, pain attacks, and numbness. At V2 and/or V3, significant reductions were observed for all symptoms, except for pressure-evoked pain (**Figure 1C**).

In addition to assessing the sensory symptoms of neuropathic pain using the painDETECT^®^ questionnaire, participants were also asked subjectively about individual symptoms of neuropathy during the visits. Consistent with the results of the painDETECT^®^-based assessment, paresthesia (which includes tingling sensation) and numbness were most reported, with 71.2% and 86.3% of patients reporting them at V0 (**Table 1**). At V3 up to one third of the patients reported a noticeable improvement or disappearance of sensory symptoms (not presented). Importantly, the results from both the painDETECT^®^ questionnaire as well as from the additional inquiries about neuropathic symptoms demonstrate that numbness, a primary negative sensation, improves during HCCP treatment. This is consistent with the results of a large observational study of various peripheral neuropathic pain conditions following HCCP treatment, which also revealed a decrease in numbness alongside other sensory symptoms (32, 47).

The present study showed a significant reduction in pain intensity from baseline at each time point for the last 24 hours (**Figure 3A**) and 4 weeks as calculated by the NPRS. Subgroup analysis revealed that the reduction in pain from baseline was consistent across different durations of pre-existing neuropathic pain. Irrespective of pain duration, categorized into groups of ≤ 6 months, 6 months to 2 years, and > 2 years, patients consistently reported a significant decrease in pain intensity compared to V0. In general, existing data support early treatment, but therapy may also be effective after prolonged pain (26, 27, 47, 56). In a recent comprehensive chart review study, it was found that the effect of HCCP treatment in patients experiencing peripheral neuropathic pain following breast cancer treatment (primarily surgery, chemotherapy, and/or radiotherapy) was significantly higher when treatment was started earlier. Nearly 70% of patients experiencing pain for 6 to 12 months reported significant pain reduction. Although this proportion decreased with longer duration of pain, still about 60% for those with pain lasting 1–4 years and around 50% for those with pain lasting 5–10 years or over 10 years reported significant pain reduction (27). The study used the Clinical Global Impression of Change (CGIC) questionnaire, which included pain. In contrast, a subgroup analysis within the same study, focusing on patients with CIPN, showed that patients with a pain duration of more than two years reported a significantly better response to treatment than those with a pain duration of less than two years (26). These results and those of the present study suggest that the duration of pain appears to have less influence on effectiveness in CIPN compared to other forms of peripheral neuropathic pain (56).

Evidence from randomized trials (28, 30, 36, 57) and real-world studies (26, 27, 29, 58) collectively indicates that repeated treatment with HCCP may yield sustained or even enhanced efficacy. A thorough *post-hoc* analysis of two prospective studies involving multiple HCCP administrations in patients with painful diabetic peripheral neuropathy (30, 36) and various non-diabetic peripheral neuropathic pain conditions (28) supports a progressive response pattern and underscores the benefit of repeated treatment in those who initially fail to achieve clinically meaningful pain relief of at least 30% (31). Bienfait et al. found significantly greater effectiveness of HCCP in CIPN patients who received at least three treatments compared to those who received up to two treatments only (26). Similarly, Dupoiron et al. showed that breast cancer patients with peripheral neuropathic pain initially unresponsive to HCCP, benefited from additional treatments. Of those who did not respond to the first application, 56.4% received at least one additional treatment, with 63.6% experiencing significant pain relief (27).

In this study, we observed a gradual increase in response rates over time. The proportion of patients experiencing at least a 30% improvement in pain intensity rose from 36.2% after the initial treatment (V1) to 43.5% after up to two treatments (V2). Likewise, the proportion of patients with at least a 50% improvement increased from 15.5% at V1 to 32.5% after up to three treatments (V3). These findings indicate not only sustained pain relief, but also further improvement in pain relief with repeated treatments (**Figure 3C**). It is important to note that not all patients with values available at V3 received HCCP treatment at all follow-up visits. Nevertheless, a progressive increase in pain relief due to repeated treatments was confirmed when analyzing patients who received a total of 3 treatments at V0–V2, i.e. every 3 months as per study protocol (**Figure 3B**). This trend contributed to a steady increase in the number of patients experiencing pain reductions of ≥ 30% and ≥ 50% with each treatment (**Figure 3D**).

Using the disease-specific QLQ-CIPN20 questionnaire, the present study demonstrated a significant improvement in CIPN-associated QoL compared to baseline from 4 weeks onwards until the end of the observation period. The total score improved significantly from week 4 to the end of the observation period and included improvements in sensory and motor subscales at all time points and autonomic subscales at T2 and V2. It is worth highlighting that the minimum clinically relevant difference of 2.5–5.9 for the sensory subscale and 2.6–5.0 for the motor subscale (59) was achieved by patients at all visits (**Table 2**; **Figures 4B, C**).

The population-weighted Health Utility Index (EQ-5D) significantly increased at V3 with a change of 0.15, which is twice the minimum important difference of 0.074 (60). In addition, according to the EQ-VAS, patients rated their current health status as significantly improved (**Table 3**).

As an additional indicator of the effectiveness of HCCP, which could potentially affect quality of life and functionality, the proportion of patients requiring concomitant pain medication was monitored throughout the study. The use of co-administered analgesics showed a proportional decrease from 35.6% at baseline to 26.1% and 30.0% at V2 and V3, respectively. A recent study investigating the effect of repeated administrations of HCCP on concomitant pain medication in a clinical setting found a statistically significant reduction in the average daily dose of opioids in patients who received at least two treatments, while no significant changes were observed in the daily dose of anticonvulsants (pregabalin, gabapentin). The authors point out that the decrease in gabapentin dose, although not statistically significant, could be of clinical relevance to patients, and that pregabalin is often retained due to its sleep-inducing properties (58). CIPN patients in the present study mainly received anticonvulsants, non-steroidal anti-inflammatory drugs, and metamizole (**Table 1**), which trended towards a gradual decrease in their overall usage with repeated HCCP treatment. Nevertheless, it is to be considered that the proportion of missing data increased throughout the course of the study and the quality of the documentation was not sufficient for the assessment of a change in dose.

Among the 35 patients who discontinued the study, the primary reasons included logistical challenges, medical or health considerations (such as presence of metastases and/or starting new chemotherapy), a perception of inadequate treatment effectiveness, and application site pain. Importantly, the tolerability profile of HCCP remained consistent with its well-established profile, with the most common ADRs being pain, burning sensation and erythema at the site of application. It is noteworthy that at the end of the observation over half of the participants expressed a keen interest in undergoing the treatment again, indicating a positive perception of its benefits.

The study’s limitations, such as its non-interventional, open-label design and high dropout rate, should be acknowledged. Additionally, as the study was conducted during the COVID-19 pandemic in Germany, it is important to recognize this as a potential confounding factor. Lockdowns, restricted access to clinics, and the psychosocial burden of the pandemic, particularly for cancer patients, may have influenced both endpoints and dropout rates. In view of the limitations of our study, the dosage of chemotherapy drugs should also be considered in future studies. Further investigation is needed to understand if the dosage and regimen of chemotherapy can influence the effectiveness of HCCP therapy. A more profound understanding of this relationship may foster the development of personalized and effective treatment plans for patients.

## 5 Conclusion

The results of the this open label, non-interventional study further validate the effectiveness of HCCP in CIPN demonstrated in smaller studies (40, 41).. The observed decreases in neuropathic pain intensity and symptoms were significant and consistent with the improvements in CIPN-related QoL. In patients who received up to three treatments, 37.5% had a 50% decrease in pain intensity while the use of concomitant analgesics decreases slightly. In these patients, the final painDETECT^®^ score dropped to below 12 points (a value consistent with a low likelihood of neuropathic pain) which may be indicative of a disease modifying effect as suggested previously (41). Given its good tolerability and effectiveness, HCCP can be considered a valuable treatment option for patients with CIPN.

## Data Availability

The original contributions presented in the study are included in the article, further inquiries can be directed to the corresponding author/s.
